# A History of Surgery, from Its Origin to the Present Time

**Published:** 1782

**Authors:** 


### LONDON MEDICAL JOURNAL,

For OCT. NOV. and DECEMBER,
1782.

### SECTION I.

### BOOKS.

I. *Hiſtoire de la Chirurgie, depuis ſon origine juſqu'à nos jours*; i. e. *A Hiſtory of Surgery, from its origin to the preſent time. By* M. Peyrilhe, *Regius Profeſſor of Chemiſtry in the College of Surgery at Paris, one of the Council of the Royal Academy of Surgery, Doctor of Phyſic in the Univerſity of Toulouſe, Member of the Academy of Sciences, Inſcriptions and Belles Lettres in that city, and of the Academy of Sciences at Montpellier*. Vol. II. 4to. Paris, 1780. 838 pages.

THIS is a continuation of the Hiſtory of Surgery begun by the late M. Dujardin, and of which we gave an account in our laſt number. The generality of continuations exhibit but a faint reſemblance of the

ſpirit and manner of the firſt projectors. He who engages to complete the undertaking of another, is generally led to it by ſome accidental circumſtance. His talents and inclination are rarely conſulted, and a work to which the writer himſelf ſits down with indifference, perhaps with reluctance, can hardly be expected to excite much pleaſure or ſatisfaction in the reader. But theſe obſervations are by no means applicable to the preſent work. M. Peyrilhe has formed his ſtyle, his arrangement and mode of reaſoning ſo exactly on the model of his predeceſſor, that in reading his production we do not ſeem to miſs the original author.

The volume begins with the fifth book, in which the author delineates the ſtate of ſurgery from the reign of Auguſtus to that of Marcus Aurelius, a period of nearly two centuries, which includes its progreſs from the time of Celſus to that of Galen. Speaking of the former of theſe, Celſus, M. Peyrilhe takes occaſion, on the authority of M. Goulin, (*Memoires Litteraires*, p. 281, année 1775) to correct a paſſage in Quintilian relative to that celebrated ancient. According to this correction, inſtead of *C. Celſus mediocri vir ingenio*, as the paſſage hath hitherto been been printed, we ought to read, *C. Celſus medicus, acri vir ingenio.*

It is remarked of this period, from Celſus to Galen, that it affords not a ſingle original book on ſurgery. It produced however ſeveral celebrated ſurgeons, and hiſtory informs us that the greater number of them compoſed works that were held in conſiderable eſtimation by their cotemporaries; but thoſe works, which exiſted at the time of Galen, Oribaſius, Ætius, Paulus Ægineta, and even ſtill later, have long been loſt, and are known to us only by the titles or fragments of them preſerved in the writings of others. Notwithſtanding this diſcouraging circumſtance, our author with wonderful induſtry has brought together from Aretæus, Pliny, Soranus, Cælius Aurelianus, Moſchion, and other writers, a great ſtock of materials to illuſtrate the ſurgery of that remote period.

In reviewing Aretæus's work, *de Cauſis et Signis acutorum et diuturnorum Morborum*, the only one of his productions that has reached poſterity, M. Peyrilhe thinks he has diſcovered in it a deſcription of the inverſion of the uterus, and likewiſe of the *membrana decidua* of our celebrated anatomiſt Dr. Hunter. The former of theſe he ſuppoſes to be contained in the following paſſage,

 which which he gives in a Latin tranflation, as he does almoft all his quotations from Greek writers: "Nonnunquam integra vulva de fua fede egredi-"tur, et mulieri feminibus infidet, incredidibilis "calamitas. Sed neque infpectabilis eft uterus "... plerumque fanè hujus exitus mortem affert, "accidit enim ex abortu, et magnis concuffioni-"bus et violento partu. At fi non interficit, diu "hæ vitam producunt, non vifibilia videntes, "extra alentes uterum atque foventes." De Sign. et Cauf. diut. lib. ii. cap. 2. The commentators on Aretæus have uniformly fuppofed this paffage to be applicable only to the *prolapfus uteri*, but our author offers three reafons for thinking that it alludes rather to the inverfion of that organ: 1ft. It is fpoken of as a difeafe that moft commonly proves fatal. A prognoftic which the fimple *prolapfus* is but rarely obferved to merit; 2dly. The caufes enumerated, fuch as abortion, concuffions, and violent delivery, are applicable to the inverted rather than to the prolapfed uterus; 3dly. Aretæus is filent with regard to the means of cure, which would hardly have been the cafe had he been fpeaking of a prolapfus, whereas in the inverted uterus there is little to be done except in the firft moments, and thefe were generally loft by the ignorance of of the midwives, who in thofe days were the only perfons employed in delivery. This is the fubftance of M. Peyrilhe's arguments on this fubject. He fufpects likewife that it is to the fame difeafe, the inverfion of the uterus, that Celfus alludes in the preface to his firft book, where he fpeaks of a new difeafe, or at leaft of one with which the principal phyficians in Rome were unacquainted. His words are, " Rarius, " fed aliquando morbus quoque ipfe novus eft, " quem non incidere, manifeftè falfum; cum " ætate noftrâ quædam, ex naturalibus partibus, " carne prolapfa et arente (fome of the fcholiafts " read *hærente)* intra paucas horas expiraverit; " fic ut nobiliffimi medici neque genus mali, " neque remedium invenerint. Quos ideo nihil " tentaffe judico, quia nemo in fplendida per" fona periclitari conjectura fua voluerit; ne " occidiffe, nifi fervaffet, videretur."

It is in the following paffage of Aretæus (*de Cauf. et Sign. Morb. diuturn.* cap. xi.) that M. Peyrilhe thinks we may diftinguifh a defcription of the *membrana decidua:* " Videtur autem " nonnunquam duplicitas uteri, interius fuccin" gens tunica quando a contigua divellitur. " Geminæ namque membranæ tantum funt " differentes a tunica, hæc vero abfcedit, et

" fluxione,

"fluxione, et abortu, et violento partu, quando "ipsa secundis inhærescit. Nam cum ipsæ vi "extrahuntur, simul et uteri tunica extrahitur; "verum nisi pereat mulier, revertens eadem "tunica utero ad amussim connectitur, aut "paulum extra prominet: contegit autem femi-"nibus mulier." Our author is likewise of opinion, that Galen seems to allude to this same membrane, when, in speaking of the changes produced in the uterus by fecundation, he says, "Qui vero humor matricis asperitates illinit, "toti superficiei internæ subtensus, membrana "tenuis efficitur" (*de usu part.* lib. xxv. cap. 3.). M. Peyrilhe very candidly acknowledges, however, that without a previous knowledge of the existence of this membrane, it would perhaps be more difficult to perceive it in Aretæus than to discover it in the dead body; and he is persuaded that Dr. Hunter owes his knowledge of it solely to those singular talents for observation and discovery which he has so repeatedly and successfully displayed in his anatomical researches. "But "after all—adds our author—we shall perhaps "never have a finer opportunity for remarking, "after Galen, what an infinite number of things "we overlook in the ancients for want of know-"ing them beforehand."

The

The trachea being lengthened during inſpiration, and becoming ſhorter as the air paſſes out of the lungs, its diameter is affected by theſe different motions, and of courſe is ſmalleſt when the tube is on the ſtretch. This circumſtance, it ſeems, concerning which modern writers have been ſilent, did not eſcape the ſagacity of the ancients. Our author has found Cælius Aurelianus alluding to it in his account of the treatment of vomica, where he adviſes the patient to lie on one ſide rather than on his back, as in the latter poſition the trachea is diſtended, and conſequently more contracted, by which means the difficulty both of breathing and of expectorating is increaſed.

In analyſing the writings of Moſchion, to whom we are indebted for the firſt treatiſe on midwifery, our author takes occaſion to quote his method of treating the furor uterinus. The whole of the preſcription is curious. "Vidua, "ſi fuerit,—ſays Moſchion—ipſa manum injiciat, "et levius habebit: virgini autem ſuccurrendum "eſt ſic: fac illi ſimilitudinem virgæ naturalis "de cerâ, et nitro et cardamomo ſecundum ætatis ejus magnitudinem, ſed hæc diligenter tere "et ſubjice, ut molle fiat, et ſit ibi quandiu pati "poterit, et carebit vitio."

The

The mention of Antigonus, who is ſpoken of by Galen as a celebrated army phyſician, leads our author to inquire into the military medical eſtabliſhment of the ancients, concerning which only a few ſcattered hints have been handed down to us by hiſtorians. He thinks it probable that among the Romans each legion had its phyſician *(medicus)*. He obſerves that Vibius Rufus, phyſician to the fifth cohort, is known to us by an inſcription, as is likewiſe Ti. Claudius Julianus, phyſician to the third; and that another inſcription, found at Brixia in Lombardy, has perpetuated the memory of a third, phyſician to the ſecond Italian legion. This laſt is as follows:

L. Cali. Arriani
Medico Legionis ii.
Italic.
Qui Vixit Ann. xxxxviii.
Menses vii.
Scribonia Fauſtina
Conjugi Cariſſimo.

There is likewiſe, we are told, ſtill extant a letter written by the Emperor Antoninus to an army phyſician, conceived in theſe terms: "Cum "te medicum legionis ſecundæ adjutricis ope "dicas, munera civilia, quamdiu reipublicæ

"cauſa,

"causâ, absfueris, suscipere non cogeris. Cùm "autem abesse desieris, post finitam eo jure vaca-"tionem, si in eorum numero es, qui ad bene-"ficia medicis concessa pertinent, eâ immunitate "uteris." *(Cod. de Profess. et Medicis, titul.* 52.).

M. Peyrilhe next proceeds to inquire whether these army physicians had any fixed stipend from the state, but on this head he has nothing certain to advance. If they had been paid by the public, he thinks they would hardly have required any reward from the individuals they assisted; and yet that this was practised is very evident from what is related of the Emperor Aurelian, that he enjoined the army physicians to give their assistance in future to the sick soldiers gratis. Flavius Vopiscus, who records this anecdote, has omitted to inform us whether the Emperor made at the same time any settled provision for the physicians.

As we know but little concerning the establishment of physicians in the Roman armies, so, according to our author, we are equally unable to determine whether they had any thing like our military hospitals. Tacitus relates, that after the fall of the amphitheatre of Fidena, in the year 28, by which fifty thousand persons were either killed or dangerously hurt, the latter were carried to the houſes of the great, and were there provided with phyſicians, and every thing neceſſary for their recovery. In a word—continues the hiſtorian—they followed on this occaſion the example of the ancients, who, after great battles, were accuſtomed to receive the wounded into their houſes, and ſupport them by their care and bounty. The Spartans, according to Juſtin, (*lib.* 28. *cap.* 4.) made a ſimilar arrangement for their troops, after their defeat at Sallaſia; and a circumſtance of the ſame nature is mentioned by Livy (*dec.* 2. *cap.* 47.). Ælius Lampridius relates of Alexander Severus, that after the manner of Cæſar, Labienus, and many other Roman Generals, this Emperor uſed to viſit the ſick ſoldiers in their tents, and if their diſorders became dangerous, directed them to be placed in private families, where they might be taken proper care of at his expence.

From the above and other paſſages in the ancient hiſtorians, our author thinks it appears clearly that the Romans had no military hoſpitals, even under the Emperors, till the beginning of the CXIth century, and conſequently that they had none during the time of the Republic. With regard to the *valetudinaria*, or infirmaries mentioned by Columella, Seneca, and Tacitus, he

ſuppoſes,

ſuppoſes, with Mercurialis, that theſe were only apartments in the houſes of perſons of fortune, deſtined for the reception of their ſick ſlaves. The temple of Eſculapius he conſiders as the ſole public edifice in Rome that was open to the public in caſes of ſickneſs, and this was intended only for the reception of thoſe ſtrangers who chanced to fall ſick during thoſe celebrated games, which drew half the inhabitants of Italy to the metropolis. As for the ξενοδοχεῖον (*xenodochia*, hoſpitals) of the ancient Greeks, which were afterwards imitated by the Emperors when the ſeat of empire was transferred from Rome to Conſtantinople, our author is of opinion that theſe were never intended for the ſick. Their derivation was from ξενος, *hoſpes*, and like the caravanſeras of the preſent day in the Eaſt, they were erected merely for the accommodation of travellers. The firſt mention of a public infirmary at Rome occurs, it ſeems, in one of St. Jerome's letters, where he obſerves that Fabiola, a Roman lady, who died about the year 400, diſtinguiſhed herſelf by this kind of charity, and was the firſt who eſtabliſhed an hoſpital, or νοσοκομεῖον, for the reception of the ſick, whom ſhe collected from the ſtreets and ſuccoured with her own hands. We ſhall tranſcribe the whole of the paſſage, which is as follows: "Quin

" potius omnem cenſum, quem habere poterat,

" (erat autem ampliſſimus, et reſpondens generi

" ejus) dilapidavit ac vendidit, et in pecuniam

" congregatum, uſibus pauperum præparavit:

" et *prima omnium* νοσοκομεῖον inſtituit, quo ægro-

" tantes colligeret de plateis, et conſumpta lan-

" goribus atque inediâ miſerorum membra fo-

" veret. Deſcribam ego nunc diverſas hominum

" calamitates, truncas nares, effoſſos oculos, ſe-

" miuſtos pedes, luridas manus, tumentes alvos,

" exile femur, crura turgentia, et de exeſis,

" ac putridis carnibus vermiculos bullientes."

(*Epiſtol. lib.* 3. *epiſt.* 10.) It does not appear, however, that this inſtitution was ſupported after the death of its founder; and our author is of opinion, that public infirmaries were not permanently eſtabliſhed in Rome before the time of the Cruſades, in the twelfth century. He is willing, however, to allow a greater antiquity to the hoſpital at Lyons, the firſt foundation of which is aſcribed to Queen Ultrogotha, wife of Childebert I. who died in 558.

In examining the fragments of Philumenus, preſerved by Ætius, M. Peyrilhe has obſerved, that in deſcribing the inflammation of the uterus, he points out the ſymptoms that diſtinguiſh it from from the *retraction of the uterus*, a diseafe which he had noticed elfewhere. This part of his writings, however, it feems is loft; but this defect our author thinks is fupplied by another fragment preferved by Ætius, and afcribed to Afpafia. " This vifcus—fays Afpafia, fpeaking of the uterus—(*Ætius*, *retr*. iv. *fer*. 4. *cap*. 77.) " deviating from its natural pofition, may in- " cline itfelf in feveral different directions. The " exiftence and fpecies of mifplacement may be " difcovered by the *touch* and the following figns. " If the uterus is mifplaced obliquely, its new " fituation will be indicated by the diftenfion, " pain, coldnefs, uneafinefs and numbnefs of the " thigh adjacent: fometimes alfo this extremity " waftes, and becomes incapable of fupporting " or moving the reft of the body. If the uterus " is thrown backwards or downwards, the diffi- " culty, and fometimes the inability for motion, " feizes both thighs. In that cafe the pain is " violent, the belly is bound, the patient is " unable to break wind, and clyfters can be " thrown up only when fhe is placed on her " hands and knees. The pains increafe when " fhe fits, efpecially when the uterus is inclined " towards the anus. If the uterus inclines for- " wards, towards the hypogaftrium, the belly

" and

" and pubis become diſtended and painful, and " ſometimes the urine is altogether ſuppreſſed.

" In whatever direction the uterus is miſ-" placed, the firſt remedies to be applied are " thoſe which are required in inflammation; " but the caſe in which it is inclined downwards " towards the anus requires particular attention. " In the firſt place the midwife muſt introduce " a finger at the anus, and endeavour to puſh " back the uterus; after which ſhe muſt intro-" duce a peſſary into the vagina to ſupport it. " If the retraction is oblique, the reduction may " be effected by the vagina, where a finger in-" troduced by means of a ſpeculum, will bring " back the neck of the uterus to its proper " poſition: in the mean time, in order to favour " this effect, the patient muſt be directed to lie " either on her back, or on the ſide oppoſite to " that to which the uterus is inclined."

Our author makes no particular remark on this paſſage, which he gives in a French tranſla-tion, but contents himſelf with referring his reader to the Medical Obſervations and Inquiries, where they may compare it with the deſcription given of the retroverted uterus. We have done this, but without feeling our obligations to Dr.

Hunter

Hunter on this head at all diminished by the comparison.

Returning to the fragments of Philumenus, M. Peyrilhe observes, that he seems to have been the first who in midwifery practised turning and delivering the child by the feet, a discovery which a modern writer (*Le Roy, pratique des accouchemens*) has improperly ascribed to Moschion.

In the sixth book, which fills the remainder of the volume, our author describes the state of surgery from Galen to Paulus Ægineta, that is, from the reign of Marcus Aurelius to the taking of Alexandria by the Saracens. During this long period of nearly five centuries, the art, he observes, made no very considerable progress. "Theory founded on the philosophy of the "times; divisions and definitions, equally ne- "cessary for the acquisition as for the transmission "of knowledge; more precision and method; "a considerable number of operative processes "improved; some modes of treatment newly "invented; the *formulæ* of medicines, rather "than the medicines themselves, multiplied "even to profusion; these are all that the reader "must expect, or at least all that can be col- "lected from the writings of this period." Poor however—adds our author—as it is in invention, it it is not one of the leaſt intereſting to the hiſto-rian. It was to the writers of thoſe ages, Galen, Oribaſius, Ætius, and Paulus Ægineta, that the Arabians owed the little they knew of good ſurgery; and at the revival of letters, the Arabian ſurgery ſerved as a clue to the ſources from which it had been drawn.

From ſeveral paſſages in Galen, our author is led to ſuppoſe that he did not, as the generality of writers have imagined, derive his knowledge of anatomy altogether from brutes. M. Peyrilhe obſerves, that Seneca ſpeaks of the diſſection of human bodies as a thing practiſed in his time; and that Galen more than once points out the difference of ſtructure in the brute and human anatomy, a circumſtance which our author conſiders as a proof that he was equally well acquainted with both. Among other marks of his ſuperior knowledge in anatomy, that paſſage (*de ſymptom. cauſis*, lib. 1.) is quoted in which Galen tells us, that when the fifth vertebra is affected the hands become paralytic.

